# Impacts of Infectious Dose, Feeding Behavior, and Age of *Culicoides sonorensis* Biting Midges on Infection Dynamics of Vesicular Stomatitis Virus

**DOI:** 10.3390/pathogens10070816

**Published:** 2021-06-29

**Authors:** Paula Rozo-Lopez, Berlin Londono-Renteria, Barbara S. Drolet

**Affiliations:** 1 Department of Entomology, Kansas State University, Vector Biology Laboratory, Manhattan, KS 66506, USA; paularozo@ksu.edu; 2 Arthropod-Borne Animal Diseases Research Unit, Agricultural Research Service, United States Department of Agriculture, Manhattan, KS 66502, USA

**Keywords:** vesicular stomatitis virus, *Culicoides* midges, infectious dose, blood-feeding behavior, vector age

## Abstract

*Culicoides sonorensis* biting midges are biological vectors of vesicular stomatitis virus (VSV) in the U.S. Yet, little is known regarding the amount of ingested virus required to infect midges, nor how their feeding behavior or age affects viral replication and vector competence. We determined the minimum infectious dose of VSV-New Jersey for *C. sonorensis* midges and examined the effects of multiple blood-feeding cycles and age at the time of virus acquisition on infection dynamics. A minimum dose of 3.2 logs of virus/mL of blood resulted in midgut infections, and 5.2 logs/mL resulted in a disseminated infection to salivary glands. For blood-feeding behavior studies, ingestion of one or two non-infectious blood meals (BM) after a VSV infectious blood meal (VSV-BM) resulted in higher whole-body virus titers than midges receiving only the single infectious VSV-BM. Interestingly, this infection enhancement was not seen when a non-infectious BM preceded the infectious VSV-BM. Lastly, increased midge age at the time of infection correlated to increased whole-body virus titers. This research highlights the epidemiological implications of infectious doses, vector feeding behaviors, and vector age on VSV infection dynamics to estimate the risk of transmission by *Culicoides* midges more precisely.

## 1. Introduction

*Culicoides* biting midges (Diptera: Ceratopogonidae) are significant agricultural pests and biological vectors of orbiviruses, orthobunyaviruses, bunyaviruses, and rhabdovi-ruses [1,2]. Specifically, in the U.S., *Culicoides sonorensis* midges play a critical role in the epidemiology of the rhabdovirus, vesicular stomatitis virus (VSV) [3,4,5,6,7,8,9,10]. Vesicular stomatitis (VS) is a viral disease of cattle, horses, sheep, goats, llamas, alpacas, and domestic and feral swine. Clinical disease includes excessive salivation and vesicular lesions of the gums, tongue, naso-oral mucosa, teats, and coronary bands, and is indistinguishable from foot-and-mouth disease in cattle and swine [11]. Economic losses are due to animal health effects, but most significantly from animal movement restrictions and quarantine measures. The epidemiology of VS is complex, involving multiple animal species, insect vectors, and routes of infection, requiring significant resources to monitor and predict outbreaks. While endemic in tropical and subtropical regions of the Americas [12], outbreaks in the U.S. with two main serotypes, VSV-New Jersey (VSV-NJ) and VSV-Indiana (VSV-IN), are sporadic occurring every 5–10 years. Incursions result from the northward movement of specific viral lineages from endemic regions of Central and Northern Mexico when ideal ecological conditions exist [5,11,13,14].

Vector-borne disease transmission dynamics depend on virus–vector interactions, namely the ability of the vector to replicate and transmit virus, and vector–host interactions, namely the frequency with which the vector encounters a susceptible animal for blood-feeding. Although adult *Culicoides* females rely on the consumption of plant carbohydrates as an energy source, they ingest blood to obtain protein for egg-laying [15]. Female swarms opportunistically feed on a wide range of hosts every 3 to 5 days; however, most vector species preferentially feed on domestic and wild ruminants and on horses [16]. As is characteristic of pool-feeders, *Culicoides* midges use their mouthparts to cut the epidermis and ingest blood and potentially other skin surface contaminates that pool in the wound. This causes significant mechanical damage to the dermis and induces physiological and immunological responses favorable for rapid infection and systemic dissemination of arboviruses delivered during blood-feeding [17]. 

Infectious VSV particles have proven difficult to detect in the blood of infected animals with viral RNA readily detected in plasma or serum (RNAemia), possibly due to the action of the vertebrate complement system or other blood factors that interfere with laboratory virus isolation methods [18]. Vesicular lesions and saliva contain high, measurable virus titers, and contaminate skin surfaces where midges pool-feed [19]. For VSV acquisition, midges must feed on infected hosts that are shedding virus at titers high enough to be taken up in their 100–150 nL volume blood meal (BM) [20,21]. Ingested virus must survive the digestive environment of the midgut, infect the midgut epithelium, and result in progeny virus crossing the basal lamina layer to disseminate into the hemolymph and subsequently infect and replicate in surrounding tissues, including the salivary glands. The number of virions ingested during a BM likely determines the success of overcoming the vector’s intrinsic immunological and physical barriers and influences the overall vector competence [22]. Thus, the number of infected midges resulting from a single swarm-feeding event depends on the viral load of the shedding animal, the efficiency of viral uptake, and intrinsic events within the vector. 

The vector competence of *C. sonorensis* midges has been previously investigated in laboratory studies by providing a high titer single infectious BM to newly emerged females [10,23]. However, this reference scenario may underestimate the epidemiological importance of vector feeding behaviors, age, and blood meal infectious doses. The magnitude of ingested titers and the effects on viral replication rates and subsequent bite transmission are important parameters to determine vector competence and overall vector capacity [22,24]. Previous work in mosquitoes has shown a virus titer threshold requirement in the initial blood meal for successful viral replication in the midgut and further dissemination [22,25]. In most cases, the proportion of infected mosquitoes positively correlates with the virus dose ingested in artificial blood meals [26,27]. Although viremia titers and duration are unknown for clinically infected animals, vector species can become infected with low or undetectable viremias [28]. Therefore, it is necessary to explore the impact of VS viral doses on midge infection rates and transmission potential.

The gonotrophic cycle (GC) comprises the time from the ingestion of a BM to the egg-laying event. Under natural conditions, *Culicoides* midges will feed multiple times during their three to six week lifespan sustaining multiple cycles [29]. The blood-feeding process itself influences pathogen amplification and dissemination in other vector species. In mosquitoes, every blood meal triggers physiological changes, such as mechanical distention of the midgut that induces apoptosis and regeneration of midgut epithelial cells [30,31,32] and may enhance the probability of viral infection. Thus, successive feeding cycles may alter the midgut permeability and enhance or accelerate virus dissemination [30,31,32]. Moreover, each blood meal also alters innate immune responses in mosquitoes, influencing the likelihood of pathogen replication [33,34,35]. Age-related decline in the immune responses, including levels of melanization [36,37], the number of circulating hemocytes [38], and the overall phagocytic capacity [37,38], may contribute to increased mortality of older mosquitoes after a pathogenic challenge [37,38]. As *Culicoides* midges share many biological traits with mosquitoes, midges acquiring a VSV infection during their first blood meal may have increased potential for transmission through feeding-related and age-related mechanisms that favor virus amplification [39]. Therefore, investigating the impact of age and additional blood-feeding on midge infection are critical to estimating *Culicoides*-VSV transmission dynamics accurately. 

## 2. Results

### 2.1. Effect of Infectious Dose on C. sonorensis VSV Infection and Dissemination Rates

Six serially diluted viremic blood meals ranging from 3.2 to 8.2 log_10_ plaque-forming units (PFU) per mL were provided to *C. sonorensis* females. Immediately after feeding, the ingested virus titer was measured in individual fully engorged midges by plaque assay and cytopathic effect (CPE). All females fed to repletion on each of the six viral dilutions were positive for infectious virus as detected by CPE. However, quantitation of virus titers by plaque assay was only achieved for the higher infectious dose groups (Table 1). As expected, the mean titer of ingested VSV was highly correlated to infectious dose with the lowest detectable ingested mean titer of 1.7 logs PFU/mL in whole-body homogenates (Table 1; Figure S1). 

**Figure 1:**
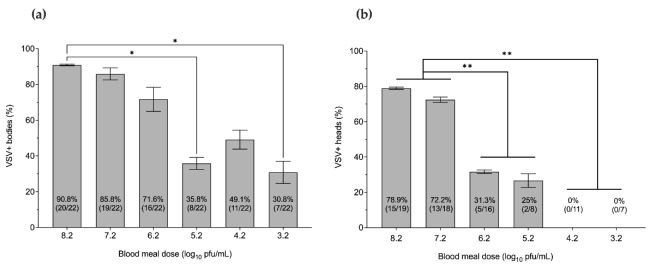
Effect of VSV blood meal infectious dose on midge midgut infection and dissemination rates at 10 days post-feeding as detected by RT-qPCR. (**a**) Proportional infection rates based on RNA detection in individual bodies. (**b**) Proportional dissemination rates based on RNA detection in individual heads with salivary glands. Statistical significance was determined by Kruskal–Wallis for multiple comparisons (* *p* ≤ 0.05; ** *p* < 0.01). Error bars represent the standard error of the mean (SEM).

Ten days after feeding on an infectious meal, bodies and heads with salivary glands of individuals were assayed separately by RT-qPCR to determine midgut infection and dissemination for each infectious dose, respectively. Decreased rates of midgut and disseminated infections were detected with decreasing titers of the infectious VSV blood meal (VSV-BM). The minimum VSV-BM infectious dose to infect midge midguts (30.8%) was 3.2 log_10_ PFU/mL (Figure 1a). The minimum infectious dose to result in a disseminated infection to heads and salivary glands (25%) was 5.2 log_10_ PFU/mL (Figure 1b).

**Figure 2:**
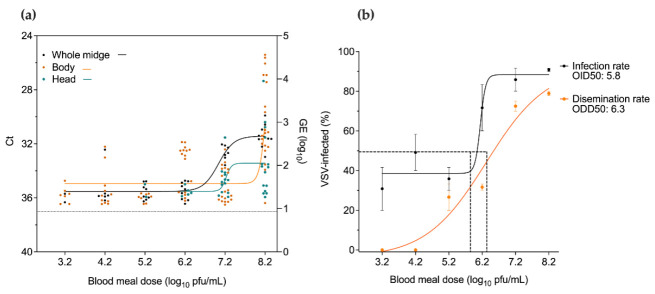
Logistic models for VSV infection and dissemination in midges at 10 days post-feeding. (**a**) RT-qPCR cycle threshold values (left Y-axis) and calculated log_10_ viral genome equivalents (right Y axis). Sigmoidal 4PL model (GraphPad) was used to perform the dose–Ct curve analysis (R^2^ of whole midge = 0.83, body = 0.50, head = 0.31). (**b**) Non-linear regression model of the oral dose vs. the mean percentage of infection indicating the oral infectious dose (OID50, R^2^ = 0.84) and oral dissemination dose (ODD50, R^2^ = 0.95).

To further analyze the minimum infectious dose-response, a non-linear logistic regression model, using cycle threshold (Ct) values, was used to estimate VSV dose-response for midgut infection (bodies) and dissemination (heads with salivary glands) 10 days after ingesting a VSV-BM (Figure 2a). Virus titers of bodies showed an exponential increase in infection with increasing oral infectious doses with the highest titers observed among the highest oral dose provided. The non-linear Ct curve for individual heads was used to estimate a VSV disseminated infection (i.e., potential transmission) dose-response. Virus titers of heads showed a logistic growth as a response to the oral infectious doses. Head titers reached a threshold (i.e., increased potential transmission) with oral doses above 7.2 log_10_ PFU/mL. Additionally, whole-body Ct values were analyzed to determine their best fit to the exponential curve of the bodies to estimate infection or of the heads to estimate dissemination. Regression analysis indicated that the best-fit model for whole-body Ct followed the logistic curve of heads with a steeper slope from growth to plateau phase after ingesting BM titers above 7.2 log_10_ PFU/mL (Figure 2a). The steeper growth for the whole-body logistic curves is most likely due to the abrupt increase in virus quantities seen in individual bodies of midges fed with 8.2 log_10_ PFU/mL of VSV (Figure 2a). A non-linear regression model analysis was also used to calculate the oral infectious dose required for VSV to infect 50% of the bodies (OID50) and the oral infection dose required for VSV to disseminate in 50% of heads (ODD50) [40]. The detected OID50 was 5.8 log_10_ PFU/mL and ODD50 was 6.3 log_10_ PFU/mL (Figure 2b).

### 2.2. Effect of Subsequent or Prior Non-Infectious Blood Meals on C. sonorensis VSV Infection Rates and Titers

**Figure 3:**
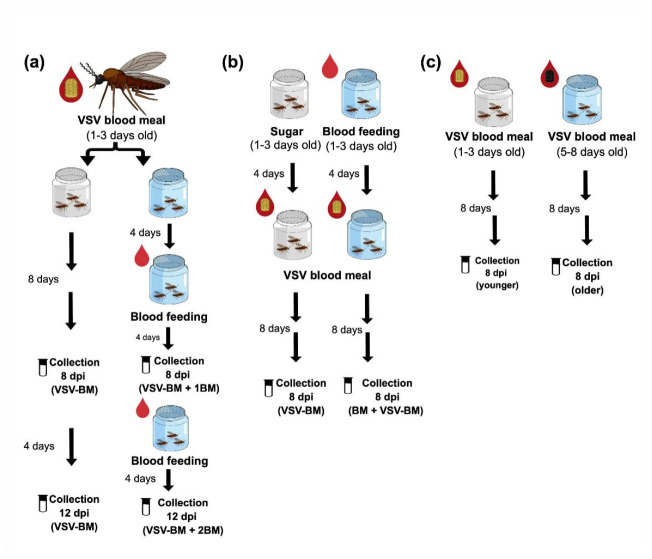
Experimental design to test the effect of (**a**) subsequent non-infectious feedings after oral infection, (**b**) VSV infection delivered as first or second blood meal, and (**c**) *C. sonorensis* age on VSV infection rates and viral titers.

**Figure 4:**
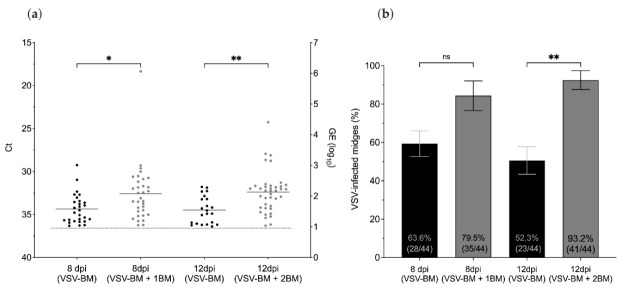
Effect of subsequent non-infectious blood meals (BM) on VSV titers and infection rates in midges as detected in whole bodies by RT-qPCR. (**a**) Detection of VSV RNA in whole midges at 8 and 12 days after having a single infectious blood meal (VSV-BM), one subsequent BM (VSV-BM + BM), or two subsequent BM (VSV-BM + 2BM). Cycle threshold values (left Y-axis) and calculated log_10_ viral genome equivalents (right Y-axis) as indicated. (**b**) Proportional infection rates for each treatment group at 8 and 12 days post-feeding. Kruskal–Wallis and multiple comparisons test used to determine statistical significance as indicated (*p* > 0.05, ns, not significant; * *p* ≤ 0.05; ** *p* < 0.01). Error bars represent the standard error of the mean (SEM).

**Figure 5:**
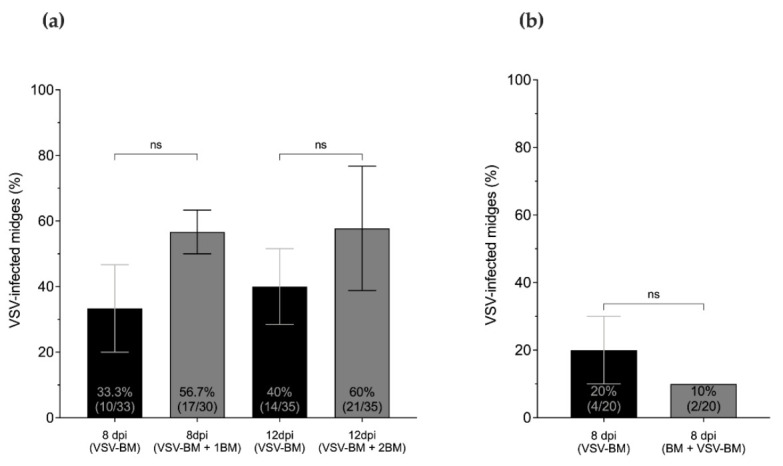
Infection rates of individual, orally infected midges as determined by cytopathic effect (CPE) screening of whole-body homogenates after two passages on Vero cells. (**a**) For subsequent meals, midges provided only a single infectious blood meal (VSV-BM) were compared to those receiving either one (VSV-BM + 1BM) or two (VSV-BM + 2BM) non-infectious blood meals at 8 and 12 days post-infection. (**b**) For prior blood meals, midges provided only a single infectious blood meal (VSV-BM) were compared to those receiving one prior non-infectious blood meal (BM + VSV-BM) at 8 days post-infection. Kruskal–Wallis and multiple comparisons test used to determine statistical significance as indicated (*p* > 0.05, ns, not significant). Error bars represent the standard error of the mean (SEM).

Virus titers and infection rates of midges that fed on a single infectious blood meal (VSV-BM) were compared with age-matched midges that received one (VSV-BM + 1BM) or two (VSV-BM + 2BM) subsequent non-infectious blood meals (Figure 3a). Virus titers were significantly higher at 8 dpi in the VSV-BM + 1BM group, and at 12 dpi in the VSV-BM + 2BM group when compared to VSV-BM midges that received only the initial infectious meal (Kruskal–Wallis test; *p* = 0.028 and 0.002, respectively) (Figure 4a). Higher overall infection rates were found in midges that received two additional non-infectious meals (Figure 4b) (*p* = 0.007). Similarly, the VSV-BM + 1BM and VSV-BM + 2BM groups had a higher percentage of positive midges as detected by CPE in comparison to age-matched midges receiving only the initial infectious VSV-BM (Figure 5a); although not statistically significant (*p* > 0.05) due to high variability between individuals. Mean titers detected by plaque assay of original homogenates were 3.7 and 3.5 log_10_ PFU/mL for 8 dpi VSV-BM and VSV-BM + 1BM, respectively, and 4.6 and 4.2 log_10_ PFU/mL for 12 dpi VSV-BM and VSV-BM + 2BM, respectively.

**Figure 6:**
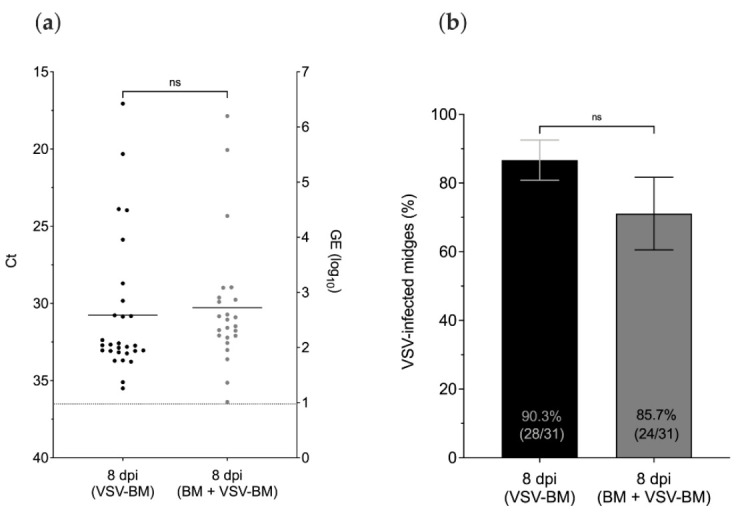
Effect of a prior non-infectious blood meal (BM) on VSV titers and infection rates of midges as detected in whole bodies by RT-qPCR. (**a**) Detection of VSV RNA in whole midges at 8 days after having a single infectious blood meal (VSV-BM) or having a non-infectious BM prior to the infectious meal (BM + VSV-BM). Cycle threshold values (left Y-axis) and calculated log_10_ viral genome equivalents (right Y-axis) as indicated. (**b**) Proportional infection rates for each treatment group at 8 days post-feeding. Mann–Whitney test was used for statistical significance as indicated (*p >* 0.05, ns, not significant). Error bars represent the standard error of the mean (SEM).

Virus titers and infection rates of midges provided one non-infectious BM prior to the infectious blood-meal (BM + VSV-BM) were compared with age-matched midges that received the infectious VSV-BM as their first meal (Figure 3b). At 8 dpi, no differences in virus titers or infection rates were observed between (VSV-BM) and (BM + VSV-BM) midges (*p* > 0.05) (Figure 6). Likewise, the proportion of VSV-positive midges as detected by CPE was similar in both groups (*p* > 0.05) (Figure 5b). Mean titers detected by plaque assay of original homogenates were 3.3 log_10_ PFU/mL for both groups.

### 2.3. Effect of C. sonorensis Age on VSV Infection Rates and Titers

**Figure 7:**
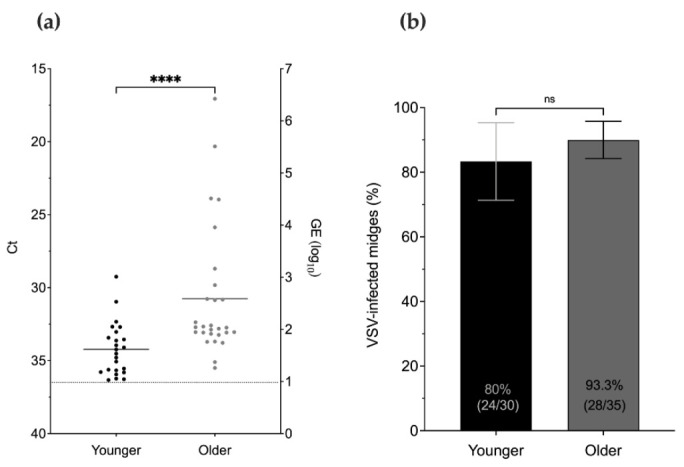
Effect of age on VSV titers and infection rates of midges as detected in whole bodies by RT-qPCR. (**a**) Detection of VSV RNA in younger and older midges at 8 dpi. Cycle threshold values (left Y-axis) and calculated log_10_ viral genome equivalents (right Y-axis) as indicated. (**b**) Proportional infection rates for each age. Mann–Whitney test analysis for statistical significance as indicated (*p* > 0.05, ns, not significant; **** *p* ≤ 0.0001). Error bars represent the standard error of the mean (SEM).

Virus titers and infection rates of midges infected shortly after emergence (younger) and midges infected 5 to 8 days after emergence (older) were compared at 8 dpi (Figure 3c). Virus titers were significantly higher in older females in comparison to younger females (*p* < 0.0001) (Figure 7a). However, there was no significant difference in the proportional infection rates between age groups (*p* > 0.05) (Figure 7b). 

## 3. Discussion

Current understanding of the VSV infection dynamics within *C. sonorensis* is limited to studies on vector competence [10,23], bite transmission [9], and non-conventional routes of transmission [6]. The physiological consequences of viral dose, feeding behavior, and age on virus–vector interactions are lacking. We determined the minimum infectious dose for *C. sonorensis* midge infection and dissemination, examined the effects of prior and subsequent blood-feeding and the effects of age on VSV infection and dissemination. These results were compared to the traditional laboratory infection methods where newly emerged *C. sonorensis* females are fed with a single, high titer infectious blood-meal (VSV-BM). 

The first determinant of vector competence evaluated in this study was the capacity of a newly emerged midge to become infected (VSV+ bodies) and have a disseminated infection (VSV+ heads) 10 days after feeding on 10-fold serially diluted infectious blood meals. Previous studies suggested that disseminated VSV infections correlate with salivary gland infection and transmission potential [10]; thus, we tested heads separate from the bodies to determine dissemination and transmission potential. We observed that higher virus titers enhance VSV infection and potential transmission while lower titers result in infections with limited dissemination, thus reducing the potential transmission. VSV+ midges were obtained for all infectious oral doses even as low as 3.2 log_10_ PFU/mL, and disseminated infections were found in midges fed on blood meals ranging from 5.2 to 8.2 log_10_ PFU/mL. Additionally, logistic modeling estimated that the probability of 50% of midges becoming infected (OID50) and becoming infectious (ODD50) by day 10 required infectious doses of 5.8 log_10_ PFU/mL and 6.3 log_10_ PFU/mL, respectively, suggesting a dose-dependent infection as observed in previous studies [22], and with higher oral doses of VSV correlating with higher infection and dissemination rates within a single gonotrophic cycle.

In VSV-infected livestock, 6 to 9 log_10_ PFU/mL are encountered in vesicular fluids, at the margins of damaged tissues, and in the copious amounts of saliva shed by symptomatic animals [19,41]. By ingesting blood in pools created within or near skin wounds, *Culicoides* midges likely ingest VSV from intact skin surfaces contaminated with saliva and vesicular sera, and from the vesicular lesions themselves. Based on our minimum infectious dose study, these reported viral levels would be adequate to initiate midgut and disseminated infections in more than 50% of midges that feed to repletion. Although high levels of viral RNA can be detected by RT-qPCR in the blood of pigs experimentally infected with VSV-NJ [18], viremia, as detected by cell culture, has not been reported in naturally or experimentally infected livestock [41,42,43,44]. However, it has been recently suggested that detecting infectious viruses by cell culture during RNAemia might be prevented by the inhibitory effect of heat-stable and thermolabile serum proteins [18]. It is unclear whether this cell culture inhibitory effect also alters the ability of the virus in the blood to infect insect midguts. Our minimum infectious dose results suggest that estimated viral levels in experimentally infected swine, based on RNAemia [18], would be adequate to initiate midgut and disseminated infections in 50% of midges fed to repletion. 

Estimating that *C. sonorensis* midges ingest a volume of 100–150 nL [20,21], feeding events on blood meals with titers lower than 5 log_10_ PFU/mL may result in fewer than one virion being ingested, lowering the chance that midges will become infected. A previous study with intrathoracic injections of *C. sonorensis* midges with 200 nL volumes of bluetongue virus at 3 log_10_ PFU/mL resulted in only 35% of midges becoming infected [45]. Thus, even when bypassing the midgut infection barrier, the inoculum is minimal at these lower concentrations that it results in approximately 65% of midges receiving no virus [28]. However, having vertebrate hosts with low viral titers does not preclude animal-to-midge transmission. *Culicoides* midges feed opportunistically in swarms, with reported feeding rates of 110 bites per minute and with the collections of 281 fed females from a single animal after only a 10-min interval [46,47,48,49]. Thus, it is expected that even at a 30.8% midgut infection rate after ingesting 100-150 nL of a meal containing 3.2 log_10_ PFU/mL VSV from an infected animal, a significant number of midges in a feeding swarm will become infected [28]. 

An additional determinant on vector competence measured in this study was feeding on non-infectious blood meals after ingestion of an infectious blood meal. Subsequent blood-feeding events have been shown to decrease the extrinsic incubation period (EIP) and increase the proportion of infectious vectors, as seen in *Leishmania*-infected sandflies [50] and in Zika- [32], dengue- [32], and chikungunya-infected [31,32] *Aedes aegypti* mosquitoes. Conversely, in *Anopheles gambiae* mosquitoes, a subsequent blood meal either did not affect the number of *Plasmodium* oocysts in terms of parasite survival and growth or negatively impacted oocyst development [35]. Our results indicate that additional blood-feeding enhances VSV replication in *C. sonorensis* midges, suggesting that successive non-infectious blood-feeding may enhance the vector’s likelihood of transmission either as a result of higher titers or potentially shorter EIPs as seen in mosquitoes, and confirming the importance of feeding behavior on vector-virus infection dynamics. Subsequent blood meals may induce extra midgut expansion and increase the number of micro-perforations in the basal lamina over time, enhancing the likelihood of virus escape [31,32]. Additional research has shown that once the arbovirus has established an infection in the vector gut, its ability to escape this barrier may not be dependent on enhanced viral replication but may be rather strongly influenced by blood-induced changes in the midgut epithelium [31,51,52]. In nature, *Culicoides* midges may ingest several blood meals to maximize the number of egg-laying cycles throughout their lifespan [15,16]. Thus, this behavior enhances pathogen transmission risk by enhancing VSV infection over time and increasing the contact frequency with animal hosts [53].

Given the potential for enhanced distention and porosity of the midgut epithelium as a consequence of blood-feeding [31,32], increased virus titers and infection rates were expected in midges that ingested a non-infectious blood meal prior to the infectious meal (BM + VSV-BM). Surprisingly, no infection enhancement was observed when virus was initially ingested in a second meal. This may be explained by evidence that blood-feeding increases circulating hemocyte numbers [53,54,55] and innate immune response activation in mosquitoes [33,35]. Blood-fed mosquitoes are able to clear more bacteria than non-blood-fed females intrathoracically challenged with *Escherichia coli* [53]. Thus, we hypothesize that *C. sonorensis* midges fed on a previous non-infected blood meal may have elevated resistance to VSV infection via an increase in the number of circulating hemocytes which are then stimulated by the second (infectious) blood meal. Thus, although bloodmeal-induced micro-perforations in midgut epithelium can facilitate the escape of viral progeny and enhance disseminated infections [31,32], these escaped viral particles face enhanced immune responses to limit viral replication [32,34,56,57]. 

Lastly, we showed that increasing midge age results in significantly higher VSV titers without altering the infection rates. Studies in mosquitoes have shown that the number of hemocytes and strength of the immune response progressively declines with age [38,54]. This immunosenescence often results in increased entomopathogenic infections in older insects [38,51]. To date, very little is known about the relationship between viral infection, aging, and immunity in *Culicoides* midges. Based on investigations of other Dipteran vectors, we hypothesize that immune-related response activation in some of the target tissues (hemocytes, the fat body, midgut) decreases with aging, allowing increased VSV replication rates. However, the effects of immunosenescence on the competence of Dipteran vectors vary between vector species and pathogen pairing. Older *Ae. aegypti* had significantly higher dengue-2 virus infection rates at early time points [52], but this phenomenon was not seen in Zika virus infection rates and titers [39]. Black flies showed decreased disseminated VSV infection with increased age [56], and older *Culex* mosquitoes and Tsetse flies are also less capable of becoming infected with parasites they vector [57,58]. Moreover, older *Ae. trivittatus* and *Ae. aegypti* are less capable of becoming infected with filarial nematodes [59,60], and older *Culex tritaeniorhynchus* mosquitoes exhibited less susceptibility to oral infection with West Nile Virus [61]. This age-related increased virus replication in midges was not seen in our previous study above when midges had ingested a prior blood meal and had gone through a gonotrophic cycle and were, therefore, older when fed an infectious meal. This lends further evidence that a prior blood meal increases innate immune responses to dampen what would have been enhanced viral replication in the older midges. Without consideration of potential alterations to vector competence and EIP dynamics, a vector’s age at the time of pathogen acquisition is a powerful driver of reduced or enhanced likelihood of transmission due to the age-dependence of daily mortality and feeding habits over their lifespan [39].

## 4. Materials and Methods

### 4.1. Virus and Cells

Stock virus (VSV-NJ; 1982 bovine field isolate, USDA-APHIS, Ames, IA, USA) was grown in porcine epithelial cells (AG08113; Coriell Institute, Camden, NJ, USA) with Eagles MEM containing Earle’s salts (Sigma, St. Louis, MO, USA) containing 2% FBS and 100 U penicillin/streptomycin sulfate at 37 °C with 5% CO_2_. Vero MARU cells (VM; Middle America Research Unit, Panama) grown in 199E media containing 2% FBS, 100 ug/mL strep, 100 units/ml Pen, and 0.25 ug/mL amphotericin B at 37 °C with 5% CO_2_ were used for detecting and titering infectious virus from midge samples as described below. 

### 4.2. VSV Infection of Culicoides sonorensis Midges

All experiments were performed using colonized *Culicoides sonorensis* midges (AK colony) maintained by USDA, Arthropod-Borne Animal Diseases Research Unit at the Center for Grain and Animal Health Research in Manhattan, KS, USA. Adult midges were maintained at 25 ± 1 °C and 75 ± 5% RH in environmental chambers with a 13:11 light: dark cycle and offered 10% sucrose solution ad libitum.

Blood meals (BM) consisted of a 1:1 mixture of defibrinated sheep blood (Lampire Biological Products, Pipersville, PA, USA) and VSV stock virus (VSV-BM; infectious meal) or sheep blood alone (BM; uninfected negative control). Midges were allowed to feed on an artificial membrane feeding system [6] for 60 min. After each meal, midges were anesthetized with CO_2_, and fully engorged blood-fed females were sorted from unfed and partially fed and maintained in cardboard cages with egg cups.

### 4.3. Effect of Infectious Dose on C. sonorensis VSV Infection and Dissemination Rates

To test the effects of infectious dose on VSV infection, 1-to-3-day-old females (3 replicates) were allowed to feed on six serially-diluted infectious VSV-BMs containing titers ranging from 3.2 to 8.2 log_10_ PFU/mL. VSV titer ingested by individual females was quantified immediately after feeding (time zero). Eight fully engorged midges from each virus dilution were collected individually in 500 µL of antibiotic medium (199E cell culture medium containing 2% FBS and 400 U/mL penicillin, 400 μg/mL streptomycin, 200 μg/mL gentamycin, 5 μg/mL ciprofloxacin, 5 μg/mL amphotericin B) and stored at −80 °C until further processing. The remaining fully engorged females were sorted into cardboard cages, maintained for 10 days, collected individually with heads separated from bodies (*n* = 22 per viral dilution) in 300 µL of TRIzol (Invitrogen, Waltham, MA, USA; Thermo Fisher Scientific, Inc., Waltham, MA, USA) for reverse transcriptase quantitative PCR (RT-qPCR), and stored at −80 °C until further processing.

### 4.4. Effect of Subsequent or Prior Non-Infectious Blood Meals on C. sonorensis VSV Infection Rates and Titers

To test the effects of subsequent BMs on VSV infection (Figure 3a), 1-to-3-day old females (4 replicates) were allowed to feed on a VSV-BM (8.2 log_10_ PFU/mL). Fully engorged females were sorted into two cardboard maintenance cages with egg cups. At 4 and 8 days, after GC-1 and GC-2, one cage was provided with a BM. As above, fully engorged females were selected and placed in new cages after each feeding. From each cage, on days 8 and 12, midges (*n* = 44 per group) were collected individually in TRIzol or antibiotic medium and stored until processing, as above.

To test the effects of a prior BM on VSV infection (Figure 3b), 1-to-3-day-old females (3 replicates) were either provided a BM or maintained on 10% sucrose. At four days, BM and sucrose-fed midges were provided a VSV-BM (8.2 log_10_ PFU/mL). Fully engorged females were sorted, maintained for eight days on 10% sucrose, sampled (*n* = 32 per group) as above, and stored at −80 °C until further processing.

### 4.5. Effect of C. sonorensis age on VSV Infection Rates and Titers

To test the effects of age on VSV infection (Figure 3c), 1-to-3-day-old females (‘younger’; 3 replicates) were provided a VSV-BM (8.2 log_10_ PFU/mL) and held for eight days. Simultaneously, a second group of 5-to-8-day-old females (‘older’; 3 replicates), that had been maintained on 10% sucrose solution, were provided a VSV-BM. Fully engorged females from both groups were selected, sorted, maintained for an additional eight days, then sampled (*n* = 32 per group) and stored until processing, as above.

### 4.6. RNA Extraction and RT-qPCR for Detection of VSV

Frozen TRIzol midge samples were thawed on ice, two 2.4 mm stainless steel beads (Omni Inc., Kennesaw, GA, USA) were added, and tubes were homogenized by shaking at 3.1m/s with a Bead Mill Homogenizer (Omni Inc.). Samples were centrifuged at 12,000× g for 6 min to pellet debris. Total RNA was extracted using Trizol-BCP (1-bromo-3chloropropane; ThermoFisher Life Technologies, Waltham, MA, USA), and RNA extracts were analyzed using TaqMan Fast Virus 1-Step MasterMix (Applied Biosystems, Waltham, MA, USA; ThermoFisher Scientific, Inc., Waltham, MA, USA) in an RT-qPCR assay detecting the L segment as previously described [6]. Standard curves and calculation of Ct values were carried out with the 7500 Fast Dx software (Applied Biosystems, Waltham, MA, USA; Thermo Fisher Scientific, Inc.). RT-qPCR reactions with Ct ≤ 36.5 were considered positive for VSV RNA [6]. To limit inter-run variations and consider the variable efficiency of each assay, a standard positive control with of known ssRNA concentration was used in every RT-qPCR assay. Cycle threshold (Ct) values plotted against the log_10_ of ssRNA VSV ng and the linear regression (y= −3.30578x+11.02683) allowed determination of viral genomic equivalents per midge [6].

### 4.7. Plaque Assays and Cytopathic Effect

To isolate infectious virus, frozen midges stored in 500 μL antibiotic media were thawed on ice and individually homogenized as above. Samples were centrifuged at 12,000× *g* for 6 min to pellet debris. Observation of cytopathic effects (CPE) after two passages was used to indicate infectious virus within that sample [6]. All homogenates with positive CPE at the first passage were further analyzed to determine infectious virus titer by standard plaque assay of the original homogenate.

### 4.8. Statistical Analysis

Data were pooled from the independent replicates of each experiment. Infection rates were calculated by dividing the number of positive midges by the total number of midges tested by RT-qPCR. Dissemination rates were calculated as the proportion of viral-RNA positive heads out of the total number of positive bodies assayed. Non-parametric tests (Kruskal–Wallis, Mann–Whitney) were used to compare Ct values and the proportion of infected midges. Non-linear regression analyses were used to evaluate the results of the oral dose experiment. GraphPad Prism version 9 (GraphPad Software Inc., San Diego, CA, USA) was used for statistical analysis and the creation of graphs.

## 5. Conclusions

This work emphasizes the importance of incorporating vector biology in understanding virus–vector interactions for VSV transmission dynamics and predictive modeling. Our results showed relatively high oral infection rates even with low blood meal titers, and enhanced virus titers in both older midges, and in midges fed on subsequent blood meals after VSV infection. Understanding these variables reinforce the ideal management practice of preventing/controlling midges before they have an opportunity to feed on a host. This research highlights the importance of considering the epidemiological implications of vector biology and feeding behavior to estimate the risk of VSV and likelihood of transmission by *Culicoides* midges more accurately.

## Figures and Tables

**Table 1:** Mean VSV titers of infectious blood meals and mean titers ingested by individual midges (*n* = 8) immediately after feeding as detected by plaque assay.

VSV-Blood Meal Titer (log_10_ PFU/mL)	Mean VSV Ingested (log_10_ PFU/mL) ^1^	Detected by Plaque Assay+ (%)
8.2	5.5	8/8 (100%)
7.2	4.1	8/8 (100%)
6.2	3.3	8/8 (100%)
5.2	2.5	5/8 (62.5%)
4.2	1.9	3/8 (37.5%)
3.2	1.7	1/8 (12.5%)

^1^ Mean ingested titer reported as PFU/mL from the 500 µL whole body midge homogenate.

## Data Availability

The data presented in this study are available on request from the corresponding authors and through the USDA Agricultural Research Information System.

## Supplementary Materials

The following are available online at https://www.mdpi.com/article/10.3390/pathogens10070816/s1. Figure S1. Correlation of mean VSV titers ingested by midges and the infectious blood meal doses on which they fed. Ingested titers were determined as individual whole-body homogenates plaqued on Vero cells.
