# Supplementary material for: Impacts of Infectious Dose, Feeding Behavior, and Age of Culicoides sonorensis Biting Midges on Infection Dynamics of Vesicular Stomatitis Virus
Source: Pathogens. 2021 Jun 29;10(7):816. doi: 10.3390/pathogens10070816 (PMC8308663; doi:10.3390/pathogens10070816)
Supplement: Supplementary file 1 [file pathogens-10-00816-s001.zip › pathogens-1257667-supplementary.pdf]

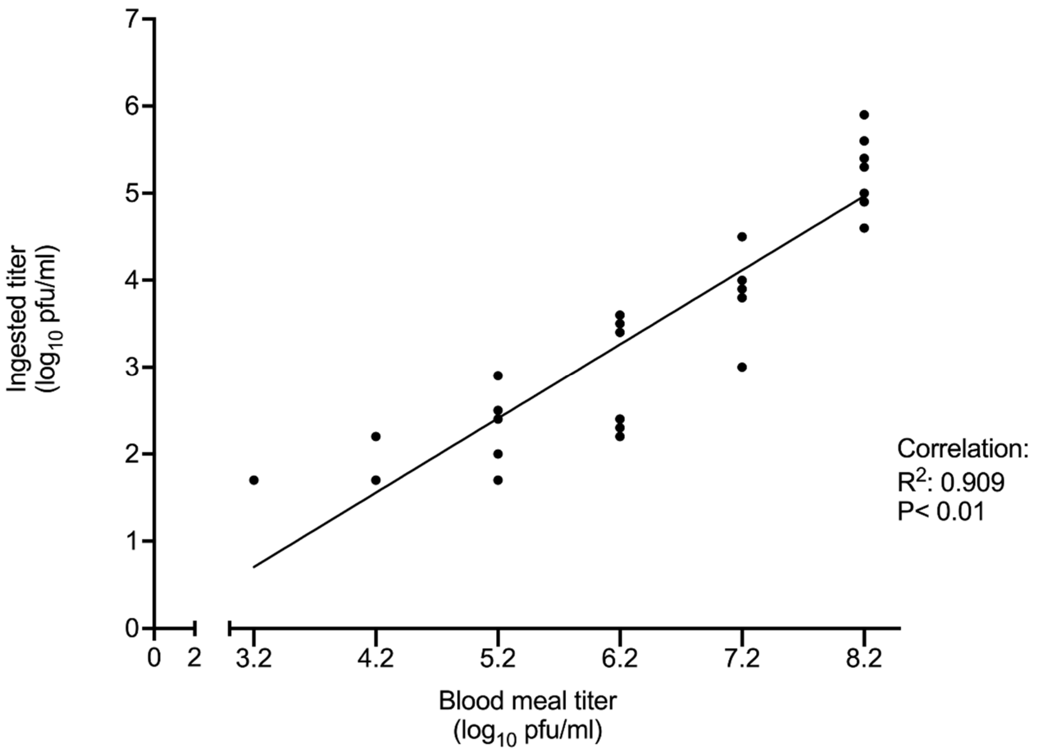

Figure S1. Correlation of mean VSV titers ingested by midges and the infectious blood meal doses on which they fed. Ingested titers were determined as individual whole-body homogenates plaqued on Vero cells.
